# The expression of signal regulatory protein alpha (SIRPα) in periodontal cells and tissue

**DOI:** 10.2340/aos.v83.41391

**Published:** 2024-09-11

**Authors:** Cecilia Koskinen Holm, Sara Rosendahl, Per-Arne Oldenborg, Pernilla Lundberg

**Affiliations:** a Department of Odontology, Section of Molecular Periodontology, Umeå University, Umeå, Sweden; b Department of Odontology, Section of Oral and Maxillofacial Surgery, Umeå University, Umeå, Sweden; c Department of Medical and Translational Biology, Umeå University, Umeå, Sweden

**Keywords:** SIRPα, inflammation, periodontal cells, periodontal tissue

## Abstract

Signal regulatory protein alpha (SIRPα) is mainly expressed by cells of myeloid origin. This membrane glycoprotein is shown to be involved in regulation of different inflammatory conditions, such as colitis and arthritis. However, SIRPα has not been investigated in relationship to periodontitis, an inflammatory condition affecting the tooth supporting tissues. We aim to investigate if resident cells in the periodontium express SIRPα and whether a possible expression is affected by inflammatory conditions. Primary human keratinocytes, fibroblasts, periodontal ligament cells, and osteoblasts were cultured with or without the pro-inflammatory cytokines tumor necrosis factor alpha (TNF-α) or interleukin-1-beta (IL-1β). All different periodontal cell types showed a basal mRNA expression of SIRPα. Pro-inflammatory cytokines induced a 2–3-fold significant increase in SIRPα expression in both cultured human gingival fibroblasts and osteoblasts but neither in keratinocytes nor in periodontal ligament cells. Tissue sections from human gingival tissue biopsies were histochemically stained for SIRPα. Epithelial keratinocytes and gingival fibroblasts stained positive in sections from periodontally healthy as well as in sections from periodontitis. In periodontitis sections, infiltrating leukocytes stained positive for SIRPα. We highlight our finding that oral keratinocytes, gingival fibroblasts, and periodontal ligament cells do express SIRPα, as this has not been presented before. The fact that inflammatory stimulation of gingival fibroblasts increased the expression of SIRPα, while an increased expression by gingival fibroblasts in periodontitis tissue *in situ* could not be detected, is indeed contradictory.

## Introduction

Our teeth are well anchored in the jawbone through a delicate tooth supporting tissue, also known as the periodontium. It includes the periodontal ligament, which is formed by the periodontal ligament cells. These anchoring fibers extend from the root surface of the tooth to the surrounding alveolar jawbone, which is formed by osteoblasts. The gingiva covers the jawbone and consists of fibrous soft tissue, which is formed and remodeled by fibroblasts. The epithelium contours the gingiva and is built up by keratinocytes.

Periodontitis is a chronic inflammatory disorder where periodontal tissue degradation jeopardizes tooth retention [1]. The inflammatory condition in the periodontium is triggered by host microorganisms that colonize the tooth surface and induce an immune response. During inflammatory processes, including periodontitis, different cytokines, chemokines, and other inflammatory mediators like prostaglandins are released, affecting and controlling the progression of the disease [2]. The pro-inflammatory cytokines tumor necrosis factor alpha (TNF-α) and interleukin-1-beta (IL-1β) are secreted from leukocytes and resident periodontal cells (i.e., gingival fibroblasts, periodontal ligament fibroblasts, and vascular endothelial cells) [3]. These cytokines play a role in periodontitis and contribute indirectly to the destruction of both connective and bone tissue, resulting in loss of tooth attachment. Both TNF-α and IL-1β enhance the expression of collagenases, and of other cytokines in cells of the periodontal lesions. Amongst those are interleukin-6 (IL-6) [4] and the cytokine receptor activator of nuclear factor kB ligand (RANKL) [5]. RANKL is crucial for differentiation and function of the bone-resorbing osteoclasts [5]. Moreover, an enhanced expression of IL-6 has been shown to stimulate the differentiation of osteoclasts [6] and may therefore also contribute to bone resorption in periodontitis. TNF-α, IL-1β, and IL-6 are found in gingival crevicular fluid from pathological gingival pockets seen in periodontitis [7, 8].

The cell surface protein signal regulatory protein alpha (SIRPα) is a protein belonging to the immunoglobulin-like super family and is highly expressed by myeloid cells [9]. In recent years, it has also been shown that SIRPα is expressed by cell types other than myeloid cells, for example, osteoblasts and stromal cells [10, 11]. There are only a few known ligands for SIRPα, where the cell surface protein CD47 is the most well described [9, 12]. The interaction between CD47 and SIRPα was initially discovered as an important “marker of self” for the host immune system to recognize endogenous from non-endogenous cells [13], as well as controlling phagocytosis [14] and formation of multinucleated macrophages [15]. Moreover, SIRPα and CD47 play an important role in leukocyte-cell migration [16] and in the differentiation of osteoclasts [11, 17]. SIRPα has also been studied in relation to inflammatory processes and conditions. It has been demonstrated that SIRPα mutant mice (lacking the intracellular signaling domain) have a reduced susceptibility to arthritis induced by type-II-collagen, hence indicating that SIRPα contributes to inflammatory tissue destruction [18]. Studies of experimental colitis in mice, and studies of the inflammatory bowel disease Crohn’s disease in humans show an accumulation of SIRPα-positive dendritic cells (DCs) in the inflamed intestine. The SIRPα-positive DCs can secrete pro-inflammatory cytokines (e.g., TNF-α, IL-6, and IL-1β). This indicates a considerable role of SIRPα in animal inflammatory models and intestinal inflammation in humans [19, 20]. However, most studies performed regarding SIRPα and inflammatory conditions have been focusing on immune- and inflammatory cells. There is limited information regarding SIRPα expression in tissue resident cells of epithelial or mesenchymal origin. Also, if inflammatory conditions would be able to change the expression of SIRPα, this could indicate a regulatory role in inflammatory tissue-destructive diseases.

Herein, we investigate the expression of SIRPα in periodontal cells and tissue under healthy and inflammatory conditions.

## Material and methods

### Cell culturing

All experiments stated below were repeated three times with at least four replicates per group.

#### Human gingival fibroblasts

Gingival fibroblasts were collected from six periodontally healthy individuals, between 22 and 25 years of age, four women and two men. The biopsies were surgically removed under local anesthesia (Xylocaine Adrenalin 20 mg/mL + 12.5 μg/mL injection fluid) from the premolar region of the mandible. The area was probed and visually examined to ensure no signs of clinical inflammation. Each biopsy measured 4 × 4 mm and was removed with a scalpel. After collection, the biopsies were dissected into smaller pieces and cultured in 25 cm^2^ culture bottles (Nunclon™ Delta Surface/ThermoFisher Scientific, USA), in alpha minimum essential medium (α-MEM), 10% fetal calf serum (FCS) (GIBCO-BRL/Life Technologies, Paisley, UK), 10 x antibiotics (Sigma Aldrich, USA) and L-glutamine (Life Technology, USA), in 5% CO_2_ at 37°C. The gingival fibroblasts migrated and proliferated from the explants and after passages 4–5 they were seeded at a concentration of 4 × 10^5^ cells/cm^2^ in 24-well culturing plates (Nunclon™ Delta Surface/ThermoFisher Scientific, USA) with α-MEM, 10% fetal calf serum, antibiotics, and L-glutamine and stimulated with IL-1β (100 pg/mL) or TNF-α (50 ng/mL) R&D Systems, USA) for 24 h in 5% CO_2_ at 37°C.

#### Human periodontal ligament cells

Periodontal ligament cells were obtained from teeth extracted on orthodontic indications from four periodontally healthy individuals, between 12 and 14 years of age. Two girls and two boys. The periodontal ligament was scraped from the middle third of the root to avoid contamination from gingival and apical tissues and cultured in 25 cm^2^ culture bottles, in α-MEM, 10% FCS, 10 x antibiotics and L-glutamine in 5% CO_2_ at 37°C. After 3–4 passages, the proliferating cells were seeded in a concentration of 4 × 10^5^ cells/ cm^2^ in 24-well culturing plates with α-MEM, 10% fetal calf serum, antibiotics, and L-glutamine and stimulated with IL-1β (100 pg/mL) or TNF-α (50 ng/mL) for 24 h in 5% CO_2_ at 37°C.

#### Human osteoblasts

Primary osteoblasts were collected from cultured trabecular bone fragments from the iliac crest extracted from six patients undergoing bone grafting procedure in spinal surgery. The trabecular bone was cut into 2–3 mm pieces, rinsed with PBS and incubated for 2 hours at 37°C with 1 mg/mL collagenase. Continued culturing was performed for 35–50 days with α-MEM, 10% fetal calf serum, antibiotics, and L-glutamine, until migration and subsequent proliferation of the cells had occurred. At passage 3, the osteoblasts were seeded at a concentration of 25 × 10^4^ cells/cm^2^ in 24-well culturing plates with α-MEM, 10% fetal calf serum, antibiotics, and L-glutamine and stimulated with IL-1β (100 pg/mL) or TNF-α (50 ng/mL) for 24 h in 5% CO_2_ at 37°C [21, 22].

#### Human oral keratinocytes

Primary gingival keratinocytes (ATCC^®^ PCS-200-014™) were seeded according to the manufacturer’s recommendations at a concentration of 5 × 10^3^ cells/cm^2^ in 24-well culturing plates with Derma Cell Basal Medium (ATCC^®^ PCS-200-030™), Keratinocyte Growth Kit (ATCC^®^ PCS-200-040™) and stimulated with IL-1β (100 pg/mL) or TNF-α (50 ng/mL) for 24 h in 5% CO_2_ at 37°C.

### RNA isolation and cDNA synthesis

mRNA was isolated using RNAqueous^®^-Micro Kit (Ambion, Austin, Texas) according to the manufacturer´s instructions. The mRNA was reversed transcribed to cDNA using High-Capacity cDNA Reverse Transcription Kit (Applied Biosystems, Foster City, CA).

### Gene expression analysis

The gene expression of SIRPα and IL-6 (ThermoFisher Scientific, USA) was analyzed with quantitative real-time RT-qPCR technique using Taq-man (API PRISM 7900HT Sequence Detection System). By simultaneous analysis of the housekeeping gene ribosomal protein L13a (*RPL13a*) (Invitrogen, USA) (Applied Biosystems, Warrington, UK), the amplification diversity of each gene was controlled. and each n/group was measured with duplicates.

### Immunohistochemical analysis

Soft tissue samples from 3 healthy individuals and 5 individuals with periodontitis were collected during oral surgery. All individuals were from northern Sweden and of Caucasian origin. The age of the individuals with periodontitis varied between 56 and 72 (56, 61, 61, 68, 72) years, three women, and two men. The healthy controls were slightly younger 55–62 (55, 59, and 62), two women and one man. All samples were collected from oral tissue adjacent to a molar. All the individuals with periodontitis had been classified with stage 3 periodontitis, exhibiting periodontal probing depth of 6 mm or more, and bleeding on probing in the area where the soft tissue was collected. The individuals with periodontitis had been treated conventionally first, with optimized oral hygiene, and removal of plaque and calculus. The samples were fixed in 4% phosphate-buffered paraformaldehyde (PFA) and embedded in paraffin. Five μm-thick tissue samples were deparaffinized in xylene and hydrated in a series of ethanol-water dilutions (99, 95, and 70%) before sodium citrate buffer heat-induced epitope retrieval and blockage with serum-free blocking solution (Dako, Denmark) were performed. The tissue samples were incubated with a polyclonal SIRPα primary antibody (SIRP alpha Antibody, ThermoFisher Scientific, USA), washed, incubated with an HRP-labelled polyclonal secondary antibody (Dako, Denmark) and after washing finally visualized using 3,3’-diaminobenzidine (DAB). A nuclear counterstaining was performed with hematoxylin. The results were photomicrographed, and the images were analyzed using Cell^B software.

### Ethical approvement

The Regional Research Ethics Committee at Umeå University approved the study. All participants gave their informed consent. The work described above has been carried out in accordance with the Code of Ethics of the World Medical Association (Declaration of Helsinki) for experiments involving humans.

### Statistical analysis

The statistical analyses were performed in GraphPad Prism 7.0a using one-way analysis (ANOVA) with Levene’s homogenicity test, and post-hoc Turkey’s test. Student’s t-test was used when appropriate. All experiments were performed at least three times with comparable results, and all data are represented as the means ± SEM. The significance levels were set to *p < 0.05, **p < 0.01, ***p < 0.001, and ****p < 0.0001.

## Result and discussion

**Figure 1:**
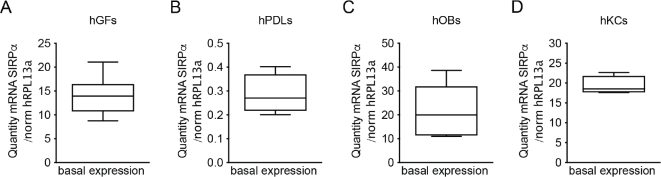
SIRPα mRNA expression in mesenchymal cells. Basal SIRPα mRNA expression in human gingival fibroblasts (A) and periodontal ligament cells (B) isolated from six and four periodontally healthy individuals, respectively, and cultured for 24 hours. mRNA expression of SIRPα in human osteoblast isolated from the iliac crest of six individuals and cultured for 24 hours (C). mRNA expression of SIRPα from primary gingival keratinocytes (D). The mRNA was quantified by quantitative real-time RT-qPCR, and the diversity of the amplification was normalized to RPL13a. Each box represents the collected mean quantity of mRNA ± SEM. hGF (human gingival fibroblasts), hPDL (human periodontal ligament cells), hOB (human osteoblasts) hKC (human keratinocytes).

In the present study, we first investigated if SIRPα is expressed in human periodontal cells by examining the expression in primary human keratinocytes, gingival fibroblasts, periodontal ligament cells, and osteoblasts. After 24 h of culturing, all cell types exhibited SIRPα mRNA expression (Figure 1A–D). The quantity of SIRPα mRNA expression was much lower in periodontal ligament cells as compared to that in keratinocytes, fibroblasts, or osteoblasts.

Periodontal cells are responsible for the maintenance of tissue structure and integrity of the periodontium [1]. However, the physiological role of these cells is not restricted to the production and remodeling of the epithelium and extracellular matrix, including the gingival fibrous tissue, periodontal ligament, and bone. Keratinocytes are shown to contribute to the inflammation in periodontitis from the early stages of infection, producing the first waves of cytokines, acting as the first signal for professional immune cell recruitment and modulation of more specific immune responses [21, 22]. This is the first report showing that keratinocytes express SIRPα but if this receptor plays a role in periodontal inflammation and tissue remodeling remains to be elucidated. Gingival fibroblasts act as sentinel cells that modulate the immune response to oral bacteria, bacterial products, and cytokines adjacent to the epithelium and gingival tissue. Thus, gingival fibroblasts act as an important “nonclassical” component of the innate immune system, by producing cytokines, chemokines, and other inflammatory stimuli [23]. As SIRPα is shown to contribute to inflammatory tissue destruction, we analyzed if cells of the periodontium alter the expression of SIRPα under inflammatory conditions [18].

**Figure 2:**
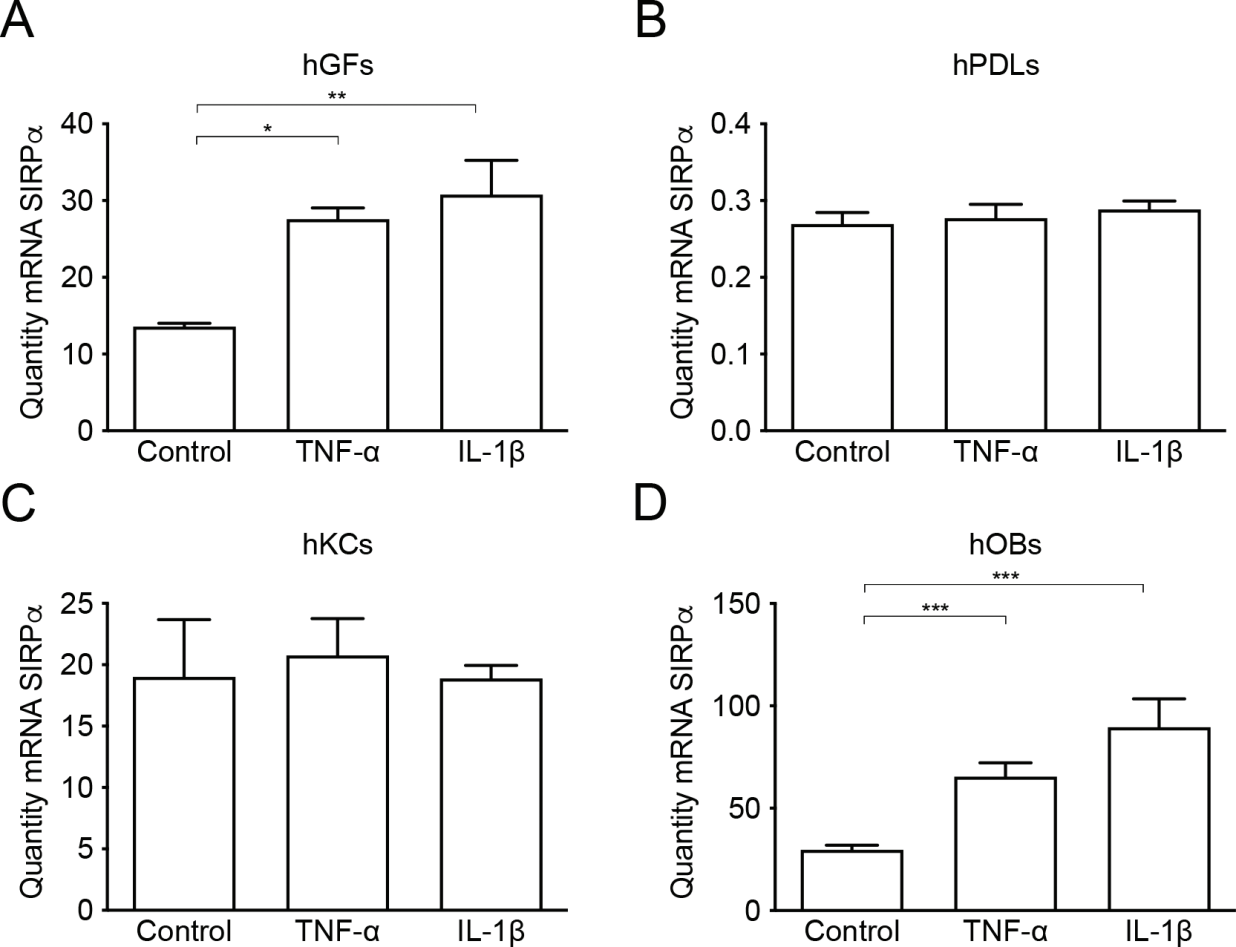
SIRPα mRNA expression in mesenchymal cells stimulated with pro-inflammatory cytokines. SIRPα mRNA expression is significantly increased in gingival fibroblasts (A) and osteoblasts (D) simulated with TNF-α (50 ng/mL) or IL-1β (100 pg/mL) for 24 h compared to control. No regulation of SIRPα mRNA expression was found in periodontal ligament cells (B) or in gingival keratinocytes (C) after stimulation with TNF-α (50 ng/mL) or IL-1β (100 pg/mL) for 24 hours. The mRNA was quantified by quantitative real-time RT-qPCR, and the diversity of the amplification was normalized to RPL13a. Data are from one representative individual per cell type and expressed as means ± SEM. hGF (human gingival fibroblasts), hPDL (human periodontal ligament cells), hOB (human osteoblasts), hKC (human keratinocytes).

**Figure 3:**
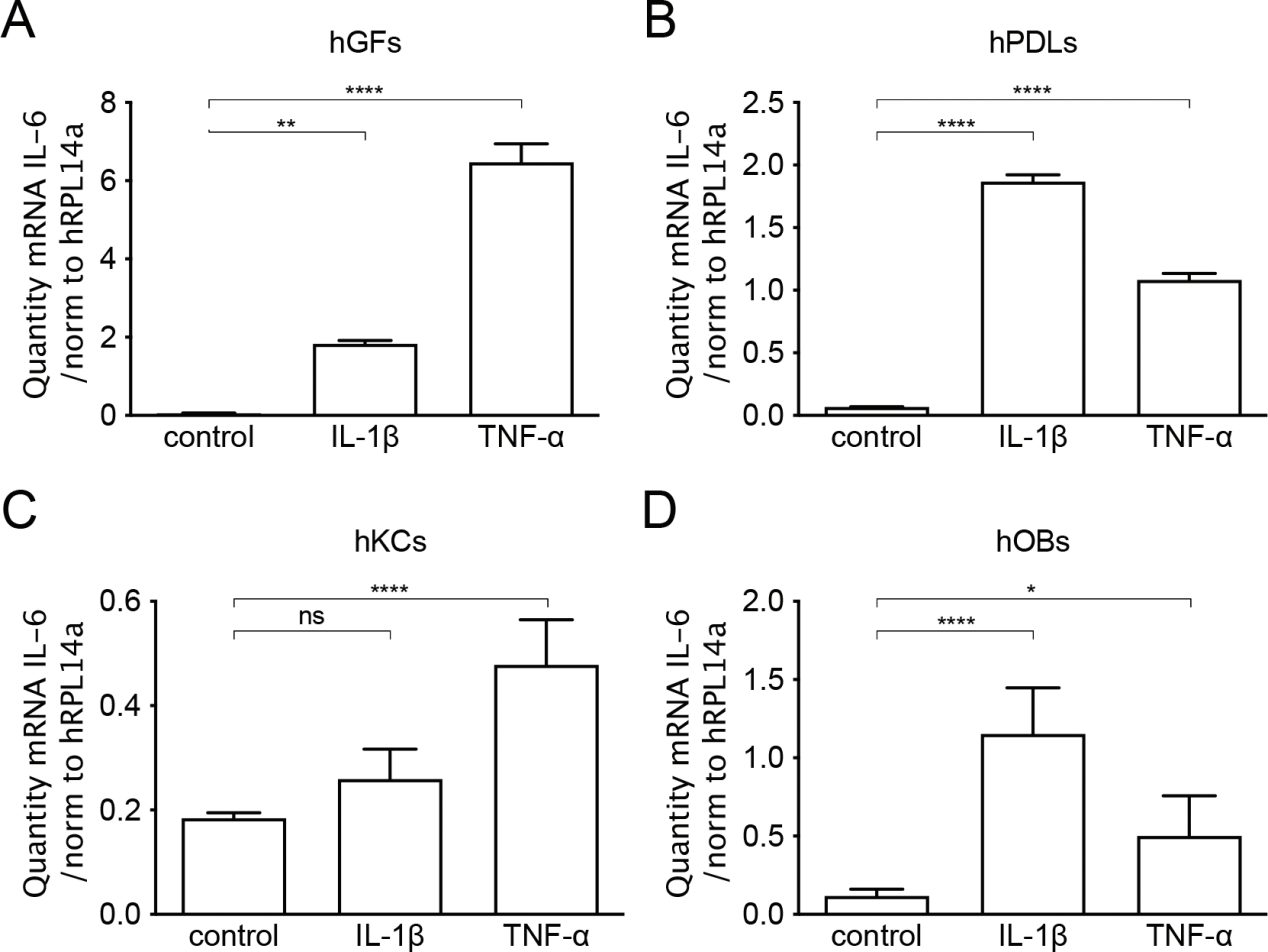
IL-6 mRNA expression in mesenchymal cells stimulated with pro-inflammatory cytokines. IL-6 mRNA expression is significantly increased in gingival fibroblasts (A), periodontal ligament cells (B), gingival keratinocytes (C) and osteoblasts (D) simulated with TNF-α (50 ng/mL) or IL-1β (100 pg/mL) for 24 h compared to control. The mRNA was quantified by quantitative real-time RT-qPCR and the diversity of the amplification was normalized to RPL13a. Data are from one representative individual per cell type and expressed as means ± SEM. hGF (human gingival fibroblasts), hPDL (human periodontal ligament cells), hOB (human osteoblasts), hKC (human keratinocytes).

The pro-inflammatory cytokines TNF-α (50 ng/mL) or IL-1β (100 pg/mL) were added for 24 hours to the cell cultures of human oral keratinocytes, gingival fibroblasts, periodontal ligament cells, or osteoblasts. In gingival fibroblasts, both TNF-α and IL-1β were able to induce a 1.9 and 2.1-fold increase of SIRPα mRNA expression respectively (*p* < 0.05 and *p* < 0.01) (Figure 2A). In contrast, addition of the selected pro-inflammatory cytokines did not change the SIRPα mRNA expression in neither periodontal ligament cells nor keratinocytes (Figure 2B, C). Like that in gingival fibroblasts, the mRNA expression of SIRPα was significantly increased in osteoblasts both by TNF-α (2,2-fold) (*p* < 0.001) and IL-1β (3-fold) (*p* < 0.001) (Figure 2D). Both IL-1β and TNF-α induced a significant increase of IL-6 mRNA expression in all the cultured cell types in comparison to unstimulated controls (Figure 3A–D), indicating a well-functioning inflammatory response in the cultures. Taken together, these data indicate that proinflammatory cytokines stimulate an increased SIRPα mRNA expression in gingival fibroblasts and osteoblasts. Fibroblasts and osteoblasts are the major cell types in the periodontium responsible for connective tissue and bone remodeling, respectively. However, it is not known if the inflammatory-related increase in SIRPα expression affects tissue remodeling in periodontitis.

The hallmark of periodontitis involves the loss of alveolar bone. Osteoblasts and bone-resorbing osteoclast, continuously and in a balanced manner, remodel the jawbone under periodontally healthy conditions. In periodontitis, unbalanced bone remodeling with increased formation and activity of osteoclasts, overriding osteoblastic bone formation, results in marginal jawbone loss. Inflammation also suppresses coupled bone formation and limits repair of the osteolytic lesions [24]. We have earlier shown a role of SIRPα, as well as its ligand CD47, in both hematopoietic cells and non-hematopoietic stromal cells, in regulating the differentiation and function of osteoclasts [11, 17]. Furthermore, our previous results showed that lack of either CD47 or SIRPα in murine bone marrow stromal cells resulted in a reduced ability to stimulate the formation of osteoclasts [11]. This makes our present findings of an increased expression of SIRPα in human periodontal fibroblasts or osteoblasts during inflammatory conditions particularly interesting. The increase in SIRPα expression could potentially be involved in stimulating the formation of osteoclasts, and thereby promoting a higher degree of bone resorption activity at the inflammatory site.

**Figure 4:**
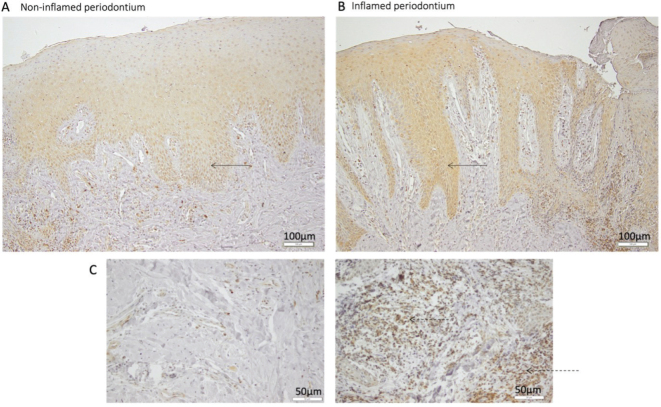
Immunohistochemical staining of SIRPα in oral tissue. Representative photomicrographs of non-inflamed and inflamed periodontal tissue, immunohistochemically stained for SIRPα. A high amount of SIRPα-positive oral keratinocytes (solid arrows) can be seen in both non-inflamed and inflamed periodontal tissue. The staining is detected in all layers of the epithelium in the inflamed periodontal tissue and in stratum basale and spinosum in the non-inflamed periodontal tissue (A, B) 10× magnification. Low expression of SIRPα in gingival fibroblasts, both in non-inflamed and inflamed periodontal tissue (A, B). A higher amount of SIRPα-positive inflammatory cells (dotted arrows) can be seen in inflamed periodontal tissue (dashed arrows) (C) 20× magnification.

To investigate whether our findings that pro-inflammatory conditions affect SIRPα mRNA expression *in vitro* also corresponds to SIRPα protein expression *in situ* in the periodontium, we analyzed SIRPα protein expression in human periodontal tissue sections. Immunohistochemical analyses of healthy gingiva revealed a positive and specific SIRPα staining of keratinocytes in the epithelium and of gingival fibroblasts in the gingival connective tissue (Figure 4A). Notable was that the SIRPα-staining of keratinocytes was foremost detected in stratum basale and stratum spinosum in the epithelium of healthy gingiva (Figure 4A). Analyses of tissue sections from individuals with periodontitis showed that the SIRPα expression was detected in all layers of the epithelium (Figure 4B). As expected, the inflamed soft gingival tissue harbored an increased number of leukocytes, and many of these cells were found to express SIRPα (Figure 4C). SIRPα positive fibroblasts were seen in both healthy and inflamed conditions. Thus, oral epithelial keratinocytes and fibroblasts express SIRPα in periodontal tissue.

We detected a higher mRNA expression of SIRPα in response to inflammatory stimuli in fibroblast cultures but found no obvious increase in SIRPα protein expression in fibroblasts of periodontitis tissues. Although the number of tissue sections from periodontally healthy and diseased tissue is too few to make any meaningful comparisons regarding differences in SIRPα expression, it is still interesting to note that the SIRPα protein extracellular domain has been shown to be sensitive to proteolytic cleavage during inflammation [25]. Such proteolytic cleave, mediated by ADAM proteases, could be induced by inflammatory agents such as PMA, microbial infection, or LPS and has been demonstrated in both myeloid cells and in human lung epithelial cells both *in vivo* and *in vitro* [25–27]. Thus, changes in SIRPα protein expression levels could be challenging to follow in inflamed tissue samples. To better understand if SIRPα protein expression is regulated in periodontitis tissues, different stages of the periodontitis disease process, from healthy tissue to severe periodontitis, need to be studied with increased numbers of participants donating tissue.

This study shows that resident cells of the periodontium and invading leukocytes in periodontitis do express SIRPα, a molecule known to play an important role in leukocyte-cell migration [16] and in the differentiation of osteoclasts [11]. Therefore, mechanistic studies are needed to further understand if SIRPα has a role in regulating periodontitis as it does in many other inflammatory conditions [18, 19, 25, 28–30].
